# A robust RMCE system based on a CHO-DG44 platform enables efficient evaluation of complex biological drug candidates

**DOI:** 10.1186/1753-6561-7-S6-P66

**Published:** 2013-12-04

**Authors:** Thomas Rose, Annette Knabe, Rita Berthold, Kristin Höwing, Anne Furthmann, Karsten Winkler, Volker Sandig

**Affiliations:** 1 ProBioGen AG, 10439 Berlin, Germany; 2 Freie Universität Berlin, 14195 Berlin, Germany

## Background

In early development stages of biologicals there is often more than one molecule against a specific target. A careful candidate evaluation is crucial to choose an optimal lead variant for further development. Complex biologicals are typically produced in CHO cells and host cells as well as the process are known to influence important molecule features such as glycan patterns or activity. To streamline the generation of stable producer cell lines we have established an Flp-based RMCE system in our CHO-DG44 platform. RMCE application allows for multi-parallel production of candidate material in the host cell and process background used for the pharmaceutical cell lines. Therefore, the molecular features of this material are expected to match with material that will be derived from a future producer cell line.

## Generation of the RMCE host cell line

A replaceable gfp gene cassette was established at random chromosomal integration sites in CHO-DG44 cells. This clone pool was subjected to a primary RMCE with a secreted and complex glycosylated alpha1-antitrypsine (A1AT) reporter. Resulting cells were screened for A1AT producers that have undergone a successful cassette exchange. This strategy allows for selection of a RMCE host cell line that combines transgene expression from highly active genomic loci with superior processing and secretion capabilities.

## Strategy for routine RMCE application

**Figure 1:**
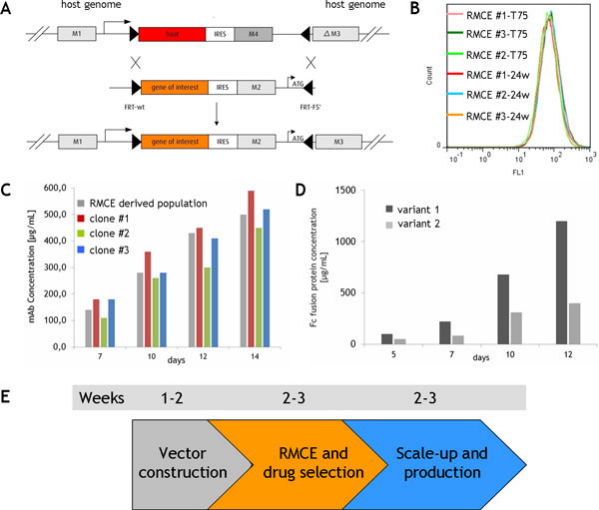
**A: RMCE Strategy for routine RMCE application in the selected CHO-DG44 RMCE host cell line**. M = selection marker, haat = A1AT gene. **B**: GFP expression of cell populations derived from multiple RMCEs and selection formats. **C**: RMCE with a monoclonal antibody. Fed batch of the direct RCME derived population and three individual RMCE clones derived from original RMCE population. **D**: RMCE with two variants of a soluble receptor-Fc fusion protein. Exemplary fed batch process for the both RMCE derived Fc fusion protein variants. **E**: Timescale of routine RMCE Application.

The selected RMCE host cell line is susceptible for cassette exchange with any desired target gene and candidate protein. Successful cassette exchange is enforced by promoter trap and a well defined selection system (Figure 1A). For RMCE application the promoterless target gene encoding for the candidate protein is cloned into a target vector where it is linked to a selection marker via an IRES element. Upon successful cassette exchange, the target and marker gene will be activated by a promoter residing at the targeting locus. In addition, a second inactive marker gene (lacking an ATG) that resides also at the host genome, but downstream of the replaceable gene cassette will be activated. The target vector is introduced together with a vector encoding the flp recombinase into the RMCE host cell line. The use of heterospecific FRT sites prevents from simple re-excision of the gene cassette.

## A robust protocol provides for efficient RMCE

RMCE application results in cell populations showing comparable expression levels of the newly introduced genes as exemplified for individual RMCEs with a gfp reporter and different selection formats (Figure 1B). Also, a homogenous expression was observed within the individual RMCE derived populations after drug selection. Efficient RMCE application is supported by a fine tuned and robust protocol that can be applied in T-flasks or multiwell formats.

## Evaluation studies: RMCE application with monoclonal antibody and fusion proteins

RMCE was applied to a monoclonal antibody and single cell clones have been generated from the RMCE derived population. Those clones were analyzed together with the original population in fed batch culture using ProBioGen's chemical defined platform medium and process (Figure 1C). The RMCE derived population yielded in harvest titers of 0.5 g/L matching the titers obtained for individual clones. Consequently, after drug selection the cells can be directly used for material production. Single cell cloning is not required!

In a second study two variants of a soluble receptor-Fc fusion protein were analyzed for manufacturability. Over a number of individual RMCEs variant #1 was expressed at a ~2-fold higher rate. In a fed batch process the difference was maintained yielding in final titers of 1.2 g/L for variant #1 (Figure 1D). The 2-3-fold outperformance of variant #1 was confirmed in classic cell line development.

## RMCE facilitates streamlined generation of stable cell lines and POC material production

At minimal effort RMCE application allows for streamlined generation of stable cell lines and production of POC material (Figure 1E). Applying a single RMCE within only 2 weeks a suspension culture is available for scale-up and production. Compared to transient protocols production runs can easily be repeated at any time and scale.

## Conclusions

A robust protocol provides for efficient and reproducible RMCE application for antibodies and single chain proteins.

At minimal effort RMCE application enables fast and multi-parallel evaluation of complex biological drug candidates.

RMCE application allows for streamlined production of candidate material in the background of ProBioGen's CHO-DG44 platform.

